# Retiform Sertoli-Leydig Cell Tumor in a 38-Year-Old Woman: A Case Report, Retrospective Review, and Review of Current Literature

**DOI:** 10.1155/2017/3421832

**Published:** 2017-02-20

**Authors:** Laura C. Nwogu, Josh A. Showalter, Suvra Roy, Michael T. Deavers, Bihong Zhao

**Affiliations:** ^1^Department of Pathology and Laboratory Medicine, University of Texas, McGovern Medical School at Houston, Houston, TX, USA; ^2^Department of Pathology and Laboratory Medicine, Houston Methodist Hospital, Houston, TX, USA

## Abstract

Ovarian sex cord-stromal tumors arise from the stromal cells that surround and support the oocytes. Sertoli-Leydig cell tumors belong to this category of ovarian neoplasms. We present the case of a 38-year-old woman who was found to have a right ovarian mass. The mass was resected and diagnosed as Stage I Sertoli-Leydig cell tumor, retiform variant, following histopathologic and immunohistochemical examination. This case is unusual given the rarity of the retiform variant of Sertoli-Leydig cell tumor and the atypically older age of 38 years at presentation.

## 1. Introduction

Ovarian sex cord-stromal tumors include pure sex cord-stromal tumors such as fibromas, thecomas, granulosa cells tumors, sclerosing stromal tumors, Sertoli cell tumors, and Leydig cell tumors. They also include mixed sex cord-stromal tumors, a category comprised of Sertoli-Leydig cell tumors (SLCTs) and sex cord-stromal tumors, not otherwise specified [1]. Malignant sex cord-stromal tumors of the ovary are rare in general, comprising only 1.2% of all primary ovarian cancers [1, 2]. SLCTs are even more uncommon, accounting for less than 0.5% of all primary ovarian neoplasms, and occur at an average age of 25 years [1, 3]. Because SLCTs are composed of cells that typically produce androgens, about half of affected patients show signs of virilization, depending on the amount of androgen production. However, virilization is less common in retiform SLCTs. The mean age at diagnosis for retiform SLCT is 15 years [1, 3]. This case report discusses the unusual case of a 38-year-old woman with a large right ovarian mass which was diagnosed as retiform SLCT following histopathologic and immunohistochemical examination.

## 2. Case Report

The patient is a 38-year-old woman who presented with a laparoscopic colostomy takedown with primary anastomosis (laparoscopic Hartmann's procedure reversal). During review of systems she endorsed vague right lower quadrant abdominal heaviness of several months duration. Physical examination of her abdomen revealed a soft, nontender, and nondistended abdomen, a small reducible parastomal hernia, and a well-healed midline abdominal scar. No other abdominal mass was documented on physical examination and no signs of virilization were noted. There were no records of gynecologic examination or abdominal imaging in the patient's electronic medical record within our system. The patient proceeded to surgery as planned for a laparoscopic Hartmann's procedure reversal. During the procedure, the patient was noted to have a large right ovarian cystic mass which was resected and sent to pathology for frozen section evaluation.

**Figure 1:**
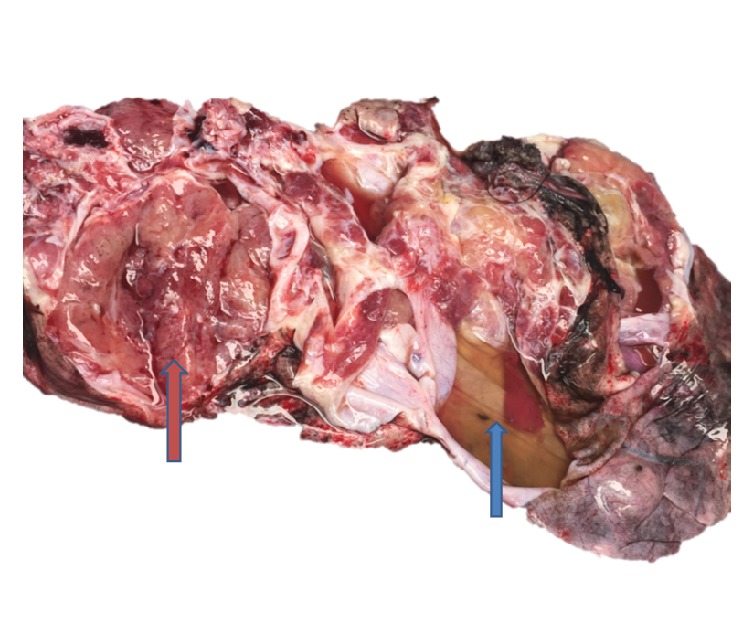
Gross image of right ovarian solid and multicystic mass which measured 27.0 × 17.0 × 5.0 cm. The capsular surface showed a 4.0 × 2.0 cm area of disruption (blue arrow). A 4.3 × 3.0 × 0.6 cm solid area was also noted (red arrow).

Gross examination showed a 27.0 × 17.0 × 5.0 cm, 727.5-gram fluctuant mass. The capsular surface demonstrated a 4.0 × 2.0 cm area of disruption. The specimen was serially cross-sectioned to reveal a multicystic tan-pink to tan-red soft cut surface with cysts ranging from 0.8 cm to 8.5 cm in diameter. Some of the cysts contained gelatinous material and the cyst walls ranged from 0.1 to 0.3 cm in thickness. Some cyst walls were focally lobulated and one cyst wall demonstrated a 4.3 × 3.0 × up to 0.6 cm tan-brown solid area (Figure 1).

**Figure 2:**
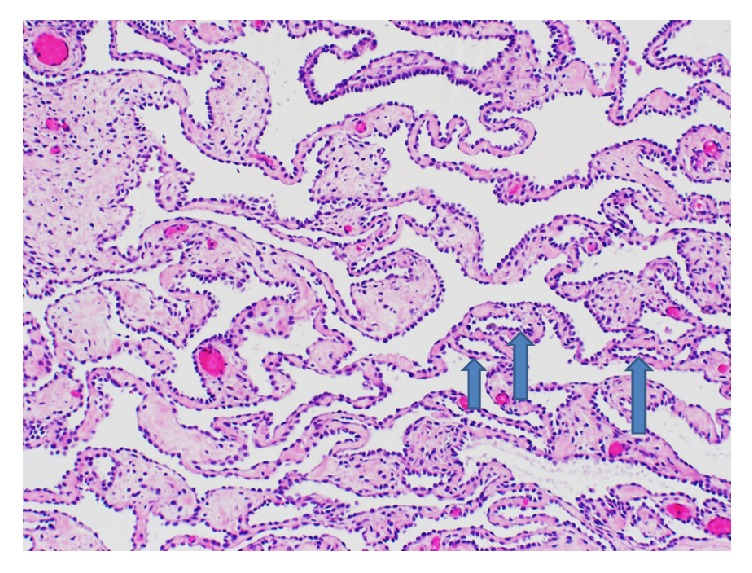
Sections from the tumor show networks of anastomosing slit-like to cystic spaces lined by cuboidal Sertoli cells, resembling the rete testis. Some of the retiform tubules are denoted by blue arrows (H&E, 100x).

**Figure 3:**
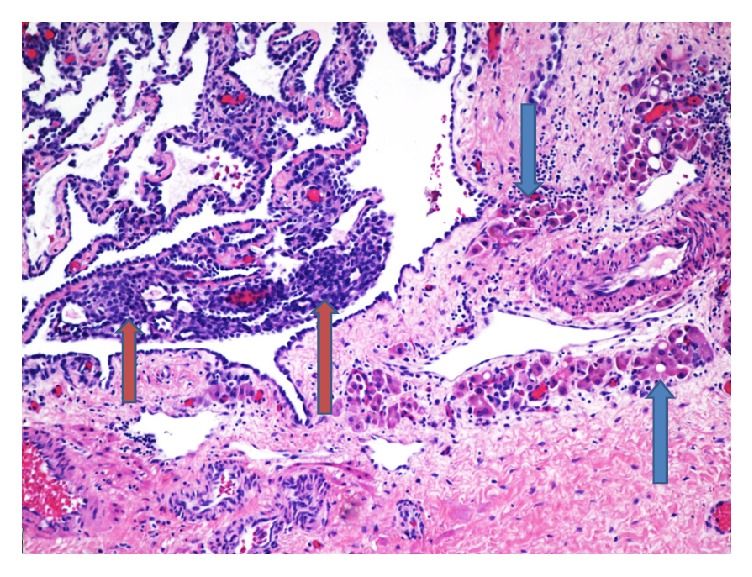
Another section of the tumor demonstrates sheets of Sertoli cells. Within the sheets of Sertoli cells, areas of small tubular formation can be seen. Clusters of Leydig cells are also noted (blue arrows) (H&E, 100x).

**Figure 4:**
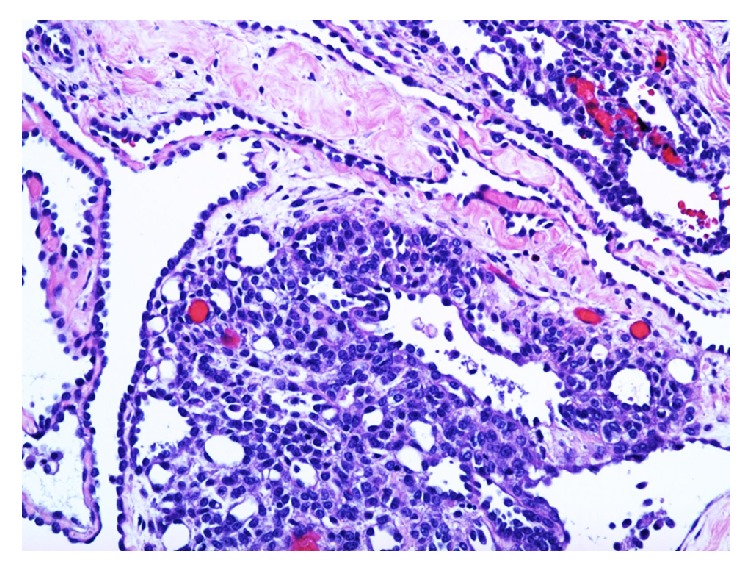
A section of the tumor shows the solid component of the tumor composed of Sertoli cells. Small tubular formation is noted in the solid component. Cuboidal Sertoli cells line the slit-like spaces (H&E, 200x).

**Figure 5:**
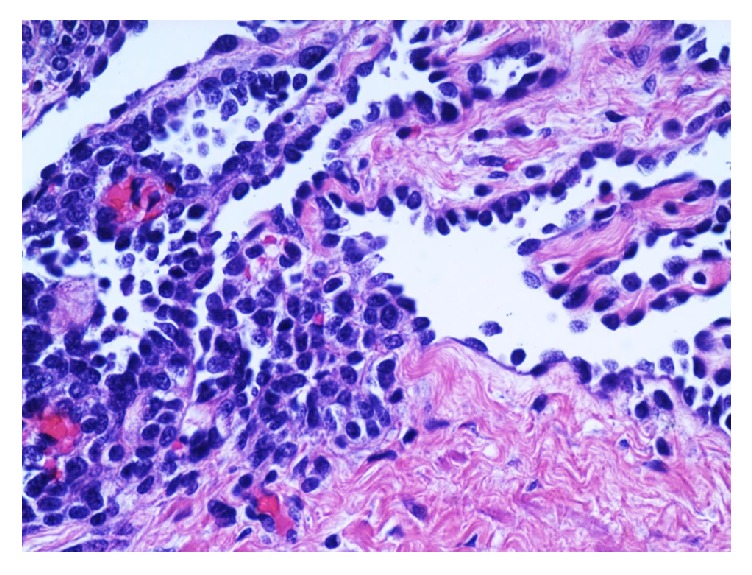
Image of the tumor demonstrating the merging of the solid component composed of Sertoli cells with the Sertoli cells lining the slit-like spaces (H&E, 400x).

Two representative sections of the right ovarian mass were submitted for frozen section analysis. The frozen section diagnosis was microcystic serous tumor, deferring to permanent sections for final diagnosis. Twenty-nine additional representative sections of the specimen were submitted for permanent sections. Microscopic examination revealed cysts with a single layer of bland cuboidal Sertoli cells and cysts with varying degrees of cellularity and fibrosis in their walls. Sections from the solid area showed focal sheets of Sertoli cells with round to oval nuclei, prominent nucleoli, and pale cytoplasm, with areas of small tubular formation. Areas with irregular networks of elongated, slit-like tubules, and papillae with fibrotic cores lined by Sertoli cells arranged in an architectural pattern resembling the rete testis were also noted in sections from the solid area (Figures 2–5). The stromal cells showed no nuclear atypia.

**Figure 6:**
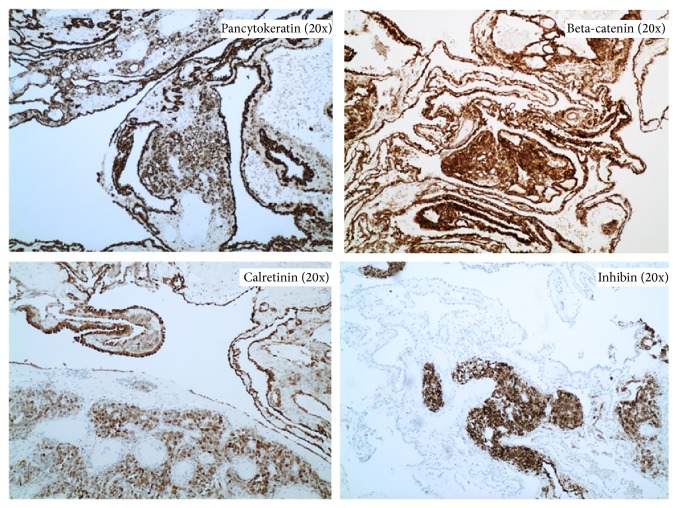
Immunohistochemical profile of the tumor for pancytokeratin, beta-catenin, calretinin, and inhibin (20x).

**Figure 7:**
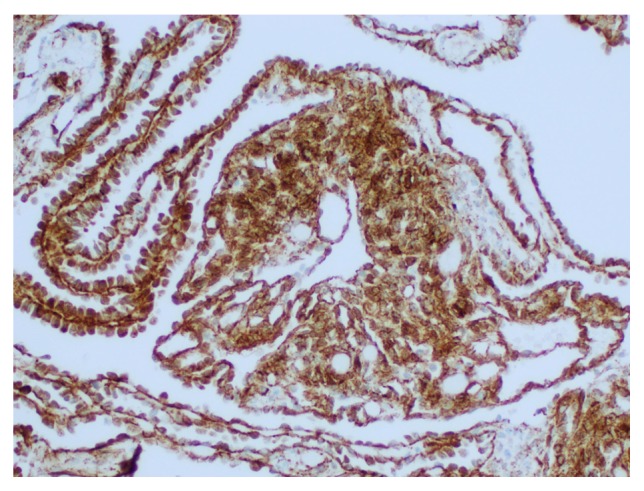
Nuclear and cytoplasmic beta-catenin positivity in retiform areas and focal solid areas composed of sheets of Sertoli cells (200x).

**Figure 8:**
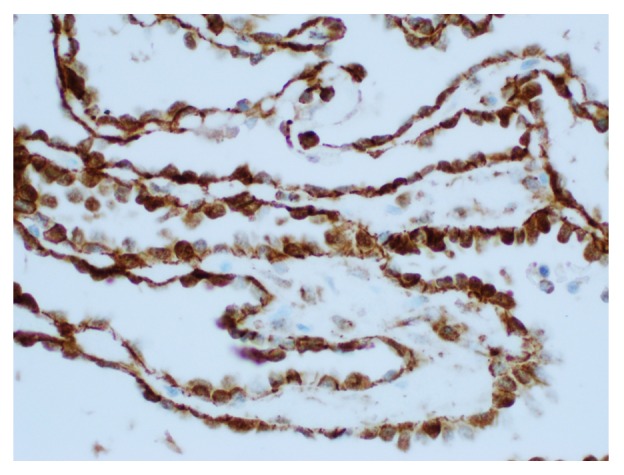
Beta-catenin with nuclear and cytoplasmic positivity in retiform areas (400x).

**Figure 9:**
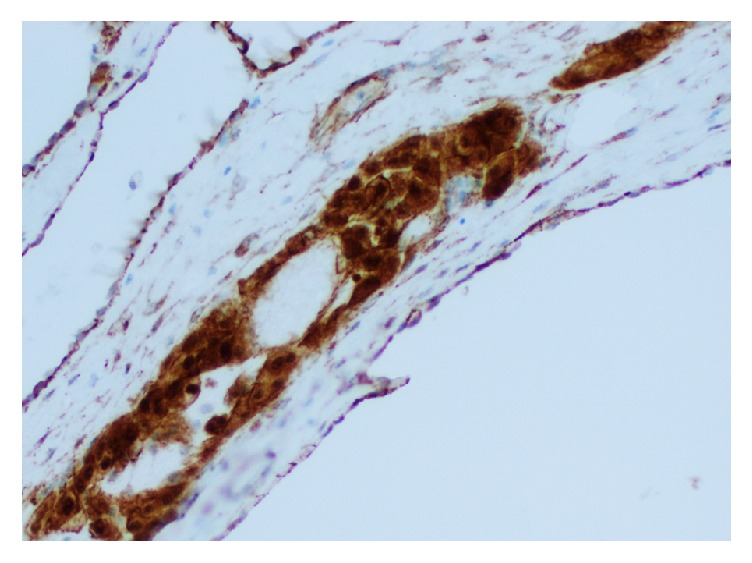
Beta-catenin with nuclear and cytoplasmic positivity in Leydig cells (400x).

Upon examination of immunohistochemical stains on sections of the right ovarian tumor, the solid component of the tumor composed of Sertoli cells was positive for pancytokeratin (strong and diffuse), beta-catenin (nuclear and cytoplasmic staining), calretinin, inhibin, WT-1 (nuclear staining), ER, and PR and negative for CK7, glypican-3, cyclin-D1, napsin, CK20, D2-40, p53, alpha fetoprotein (AFP), CD10, HEPAR-1, and arginase. The Leydig cells were positive for inhibin (strong), calretinin, and beta-catenin (nuclear and cytoplasmic) and negative for pancytokeratin, CK7, ER, PR, glypican-3, cyclin-D1, napsin, CK20, D2-40, p53, AFP, CD10, HEPAR-1, and arginase. The retiform areas of the tumor showed positivity for pancytokeratin (strong), beta-catenin (nuclear and cytoplasmic), calretinin, WT-1 (nuclear staining), PR, CK7 (weak and focal), and inhibin (weak) and negative staining for ER, glypican-3, cyclin-D1, napsin, CK20, D2-40, p53, AFP, CD10, HEPAR-1, and arginase. Images with the staining patterns of the tumor for inhibin, calretinin, beta-catenin, and pancytokeratin are presented in Figure 6. Images showing nuclear and cytoplasmic beta-catenin positivity in Sertoli cells, retiform areas, and Leydig cells are shown in Figures 7, 8, and 9, respectively. A summary of the immunohistochemical profile of the tumor by component is outlined in Table 1.

Following histologic and immunohistochemical examination by the pathology resident and pathologist, the case was further examined and discussed at the intradepartmental quality assurance consensus conference and the diagnosis of Sertoli-Leydig cell tumor, retiform variant, was agreed upon based on the morphologic and immunohistochemical findings. Additional input was sought from a gynecologic pathologist at a separate institution, who agreed with the above pathologic diagnosis.

## 3. Discussion

SLCTs are rare in general, making up less than 0.5% of all primary ovarian neoplasms [1, 3]. While SLCTs have been reported in females ranging in age from 1 to 84 years, with a mean age of 25 years at diagnosis, the average age at diagnosis for retiform SLCT is much younger at 15 years [1, 3]. A review of the literature revealed about 65 cases of retiform SLCT published in case reports and case series to date [4–18]. The patients in the published reports ranged in age from 11 months to 70 years. One case series with a total of 31 cases of retiform SLCT reported an age range of 2 to 39 years with an average age of 17 years at diagnosis. Of note, the authors initially reported 25 cases and added 6 more cases in an addendum for a total of 31 cases [15]. Another case series of 9 retiform SLCTs reported a patient age range of 11 months to 23 years at the time of diagnosis [16]. Only one distinct case report of a patient diagnosed with retiform SLCT at over 35 years of age is noted; the patient in that case was 70 years old at the time of diagnosis and presented with abdominal distention [4]. The most frequent presenting sign in the published cases of retiform SLCT was related to abdominal mass effect and included abdominal pain and increasing abdominal girth [4–18]. Endocrine manifestations such as amenorrhea and hirsutism were reported in a minority of the cases [9, 15, 16]. Only two cases of retiform SLCT were reported to be identified incidentally on routine physical examination [15].

A retrospective review of the pathologic diagnosis on all ovarian specimens signed out between June 2008 and August 2016 at our training institution was performed. A total of 963 pathology reports on ovarian biopsy, ovarian cyst wall biopsy, ovarian cystectomy, and oophorectomy specimens were identified. The specimens were collected from female patients ranging in age from 8 months to 89 years. Of the 963 pathology reports on the ovarian specimens, 426 (44.2%) represented ovarian specimens with physiologic changes or no significant histopathologic changes, and 537 (55.8%) represented neoplastic ovarian specimens. Of the 537 neoplastic cases, 415 (77.3%) were benign neoplasms, 44 (8.2%) were borderline neoplasms, and 78 (14.5%) were malignant. 68 out of the 78 malignant cases (87.2%) were primary ovarian malignancies, and 10 of them (12.8%) were metastatic tumors to the ovary. Of all the 537 neoplastic cases in this retrospective review spanning a period of 8 years and 2 months, only one case of SLCT, which also happened to be the retiform variant and the subject of this case report, was identified, representing only 0.2% of all the neoplastic ovarian specimens identified in our training institution over that time frame. These findings are consistent with the reported incidence of SLCTs in the literature and underscore the rarity of this tumor type.

The clinical features observed in patients with SLCT include androgenic manifestations in 40–60% of cases, such as the development of muscle bulk, hirsutism, deepening of the voice, amenorrhea, breast atrophy, acne, and hypertrophy of the clitoris. In fewer cases, estrogenic manifestations such as menometrorrhagia and isosexual pseudoprecocity may be noted. Patients without endocrine manifestations may present with symptoms related to abdominal mass effect such as a feeling of heaviness, abdominal discomfort or pain, ascites, or tumor rupture [2, 3, 8–11]. The patient in our case presented with health reasons unrelated to her right ovarian mass; however, upon a review of systems, she endorsed right lower quadrant abdominal heaviness of several months duration. No palpable abdominal mass or signs of virilization were noted on physical examination. The explanation for the lack of a palpable abdominal mass is unclear given a tumor size of 27 cm. Possible confounding factors include the positioning of the mass within the abdominal cavity and the patient's overweight body habitus with a body mass index of 30.8 kg/m^2^. Elevated serum AFP may be seen in rare cases of SLCT, but generally at lower serum levels than seen in yolk sac tumor [13, 19]. A serum AFP was not obtained for the patient in this case.

SLCTs are typically unilateral with approximately 2% occurring bilaterally. They range in size from 2 to 35 cm with average diameter ranging from 12 to 14 cm. They may be solid but are often partly solid and partly cystic or rarely cystic. Solid areas may appear fleshy, lobulated, tan-pink, pale yellow, or grey. Papillary excrescences may be grossly identified on the cyst walls of retiform SLCTs and areas of hemorrhage and necrosis may be seen in poorly differentiated forms [1, 3, 19, 20]. Grossly, the mass in our case was tan-pink and multicystic with focal areas of tan lobulated cyst walls, varying cyst wall thickness ranging from 0.1 to 0.3 cm, and a focal tan-brown solid area which was sampled. No areas with papillary excrescences, hemorrhage, or necrosis were noted on gross examination.

SLCTs represent a group of primary ovarian tumors in the mixed sex cord-stromal category and are composed of variable combinations of Sertoli cells and Leydig cells, among other possible components. They are divided into five histologic subtypes including well-differentiated, moderately differentiated, and poorly differentiated SLCTs, retiform SLCTs, and SLCTs with heterologous elements. The well-differentiated, moderately differentiated, and poorly differentiated forms are assigned based on the degree of tubular differentiation of the Sertoli cell component, which decreases with increasing grade, and the amount of primitive gonadal stroma presents within the tumor, which increases with increasing grade. Leydig cells also tend to decrease with increasing tumor grade [1]. Primitive gonadal stroma and heterologous elements including mucinous epithelium, striated muscle, cartilage, hepatoid cells, and rarely neuroectodermal elements may be seen in the moderately and poorly differentiated forms, in which case the tumor would be designated as including heterologous elements [1, 3, 19, 21, 22]. No heterologous elements were identified in our case.

The differential diagnoses for well-differentiated SLCT include endometrioid adenocarcinoma with sertoliform glands. The conspicuous tubules, the presence of Leydig cells, and the absence of squamous differentiation in SLCTs help to differentiate them from endometrioid adenocarcinoma of the ovary. While SLCTs characteristically show positive staining for inhibin, calretinin, and FOXL2, ovarian endometrioid adenocarcinomas are typically negative for these markers while demonstrating positivity for CK7 and EMA, which would typically be negative in SLCTs. Moderately to poorly differentiated SLCTs may be difficult to distinguish from germ cell tumors, serous neoplasms, carcinosarcomas, and the rare primary ovarian Wilms tumor. Microscopically, the cellular lobules of moderately differentiated SLCTs impart a distinctive low power appearance not typically seen in germ cell tumors. Additionally, the stromal component of poorly differentiated SLCTs demonstrates a sarcomatoid appearance, in contrast to germ cell tumors which often demonstrate a thecoma-like background. The presence of heterologous elements or a retiform growth pattern is suggestive of SLCT. While the apposition of the epithelial and stromal components of SLCT may raise suspicion for a carcinosarcoma, the typical young age at presentation, the presence of androgenic manifestations, and the identification of more typical patterns of SLCT may provide clues to the right diagnosis. Carcinosarcomas are typically negative for inhibin and positive for EMA [1, 3]. Hepatoid heterologous elements may demonstrate centrally located or eccentric nuclei with eosinophilic granular cytoplasm, resembling Leydig cells. An immunohistochemical panel of Melan-A, keratin, AFP, vimentin, inhibin, HEPAR-1, and arginase can be used to differentiate between Leydig cells and heterologous elements. The Leydig cells in our case were positive for inhibin and negative for HEPAR-1 and arginase. Intestinal type mucinous epithelium will show positive staining for CK7 and CK20 [1, 3]. A combination of clinical features, morphologic features, and immunohistochemistry is often required to facilitate the right diagnosis.

The retiform pattern is characteristically absent in well-differentiated SLCTs and is diagnosed when a significant portion of the tumor demonstrates networks of anastomosing, slit-like spaces and tubules lined by flattened to cuboidal Sertoli cells in a single layer or stratified pattern [1, 3, 19]. In one study, the criteria for inclusion as retiform SLCT were a primary ovarian neoplasm where >5% of the tissue area on the slides was composed of a retiform pattern [19]. In our case, 15 to 20% of examined representative tissue sections of the tumor had a retiform growth pattern. Other patterns that may be encountered in retiform SLCT include areas with papillary architecture and multicystic areas with slit-like spaces lined by low cuboidal to flattened Sertoli cells. The papillae may be simple or complex and cellular with branching patterns and fibrous to hyalinized cores. Stromal edema and glomeruloid structures may be encountered. The retiform pattern may be focally present in about 10% of moderate and poorly differentiated SLCTs; however only about 10% of SLCTs are purely retiform [1, 3].

SLCTs express positivity for vimentin, inhibin, calretinin, CD56, and WT-1 with varying degrees of staining intensity between the sex cord and stromal cells. Sertoli cells tend to stain less diffusely and less intensely for inhibin when compared to Leydig cells. Additionally, staining for inhibin may be weak and focal or completely absent in poorly differentiated areas of the tumor [1, 3, 23–33]. Heterologous elements show an immunohistochemical profile similar to those of their constituent tissues. In order to classify the ovarian tumor in our case, immunohistochemical stains with appropriate reactive controls were performed on representative sections of the specimen for pancytokeratin, CK7, CK20, WT-1, calretinin, D2-40, p53, AFP, glypican-3, napsin, inhibin, CD10, cyclin D1, ER, PR, beta-catenin, PAX-8, HEPAR-1, and arginase. Pancytokeratin was ordered to delineate the epithelial component of the tumor. CK7 and CK20 were obtained to assess for a gastrointestinal or genitourinary immunohistochemical profile. Intestinal type mucinous epithelium will show positive staining for CK7 and CK20 [1, 3]. AFP and glypican-3 immunostains were obtained in consideration for ovarian yolk sac tumor as part of the differential diagnosis. HEPAR-1 and arginase were added to differentiate Leydig cells from possible hepatoid heterologous elements. Leydig cells have been reported to be positive for Melan-A, vimentin, and inhibin and negative for keratin, AFP, HEPAR-1, and arginase. Hepatoid heterologous elements typically demonstrate positive staining for keratin, AFP, HEPAR-1, and arginase and negative staining for Melan-A, vimentin, and inhibin. AFP expression may also be noted in endodermal like structures [1, 3, 22]. P53, napsin, CD10, and cyclin D1 were performed in consideration for ovarian serous carcinoma, ovarian clear cell carcinoma, endometrioid adenocarcinoma, and metastatic undifferentiated endometrial stromal sarcoma, respectively, as part of our differential diagnoses.

In our case, the retiform areas of the tumor showed positivity for pancytokeratin (strong), beta-catenin (nuclear and cytoplasmic staining), calretinin, WT-1 (nuclear staining), PR, CK7 (weak and focal), and inhibin (weak) and negative staining for ER, glypican-3, cyclin-D1, napsin, CK20, D2-40, p53, AFP, CD10, HEPAR-1, and arginase. The Sertoli cells, Leydig cells, and retiform areas showed variable positivity for calretinin, beta-catenin (nuclear and cytoplasmic staining) and inhibin. The immunohistochemical findings in our case are essentially consistent with the reported immunohistochemical staining pattern in SLCT [1, 3, 23–33]. All the components of the tumor in our case, including the Sertoli cells, the Leydig cells, and the retiform areas, showed nuclear and cytoplasmic beta-catenin positivity. The significance and utility of beta-catenin has not been previously described in the literature for SLCTs. This may be the subject of future studies to delineate the various staining patterns in the different components of the tumor, across the various SLCT subtypes, and perhaps as a tool to distinguish between the various entities in the differential diagnoses.

SLCTs have an overall favorable prognosis. However, prognosis has been associated with the tumor grade and stage at presentation. About 100% survival has been reported in well-differentiated tumors. Approximately 10% of moderately differentiated tumors with or without heterologous elements were reported to be malignant. Additionally, poorly differentiated tumors, tumors with heterologous mesenchymal elements, and tumor rupture have been associated with adverse outcomes [1, 3, 19]. The presence of a retiform growth pattern is thought to confer slightly worse prognosis in cases of moderately differentiated SLCT; however, the evidence is inconclusive [1, 7, 19, 20]. For patients with well-differentiated to moderately differentiated SLCT confined to the ovary and without mesenchymal heterologous elements, unilateral salpingo-oophorectomy is the surgical management of choice. In patients with tumor rupture, extraovarian spread, poorly differentiated forms, or mesenchymal heterologous elements, a total abdominal hysterectomy followed by postoperative chemotherapy, radiation, or a combination of both is the treatment of choice [18]. In our case, the SLCT was confined to the right ovary and the patient proceeded with her planned Hartmann's procedure reversal following a right oophorectomy. In this case, no additional treatment was undertaken for the patient's SLCT after surgery.

## 4. Conclusions

This case is presented with the rarity of the tumor type and the atypically older age of 38 years at the time of diagnosis. SLCTs, including retiform SLCTs, often present a diagnostic challenge attributable to the various morphological patterns that may be assumed by the tumor. Immunohistochemistry is often required to facilitate accurate diagnosis and inclusion of inhibin is essential in a panel of stains to evaluate these tumors. Positivity for beta-catenin was noted in Sertoli cells, Leydig cells, and retiform areas in our case. The utility of beta-catenin staining for SLCTs has not been previously described in the literature. This may represent a spring board for further studies looking into beta-catenin staining patterns in the 5 subtypes of SLCT and into the utility of this marker in facilitating accurate diagnosis in higher grade SLCTs, retiform SLCTs, and SLCTs with heterologous elements. Unilateral salpingo-oophorectomy is still the treatment of choice but it remains unclear whether complete staging or postoperative adjuvant chemotherapy is necessary for the management of retiform SLCT given inconclusive evidence to support worse prognosis in the presence of a retiform growth pattern. Further studies with a large sample size and longer follow-up are needed for better prognostication and to determine if chemotherapy has a role in managing patients with retiform SLCT.

## Figures and Tables

**Table 1:** Summary of immunohistochemical profile of the tumor by component.

	Positive stains	Negative stains
Solid areas (Sertoli cells)	Pancytokeratin (strong, diffuse)	CK7
	Beta-catenin (nuclear and cytoplasmic)	Glypican-3
	Calretinin	Cyclin-D1
	Inhibin	Napsin
	WT-1 (nuclear staining)	CK20
	ER	D2-40
	PR	p53
		AFP
		CD10
		HEPAR-1
		Arginase

Leydig cells	Inhibin (strong)	Pancytokeratin
	Calretinin	CK7
	Beta-catenin (nuclear and cytoplasmic)	ER
		PR
		Glypican-3
		Cyclin-D1
		Napsin
		CK20
		D2-40
		p53
		AFP
		CD10
		HEPAR-1
		Arginase

Retiform areas	Pancytokeratin (strong)	ER
	Beta-catenin (nuclear and cytoplasmic)	Glypican-3
	Calretinin	Cyclin-D1
	WT-1 (nuclear staining)	Napsin
	PR	CK20
	CK7 (weak, focal)	D2-40
	Inhibin (weak)	p53
		AFP
		CD10
		HEPAR-1
		Arginase
